# Research on application of radiomics in glioma: a bibliometric and visual analysis

**DOI:** 10.3389/fonc.2023.1083080

**Published:** 2023-09-12

**Authors:** Chunbao Chen, Xue Du, Lu Yang, Hongjun Liu, Zhou Li, Zhangyang Gou, Jian Qi

**Affiliations:** ^1^ Department of Neurosurgery, Afiliated Hospital of North Sichuan Medical College, Nanchong, China; ^2^ Department of Oncology, The People's Hospital of Hechuan, Chongqing, China; ^3^ Department of Oncology, North Sichuan Medical College, Nanchong, China; ^4^ Department of Oncology, Suining Central Hospital, Suining, China; ^5^ Department of Neurosurgery, Nanchong Central Hospital, The Afiliated Nanchong Central Hospital of North Sichuan Medical College, Nanchong, China

**Keywords:** glioma, radiomics, bibliometrics, visual analysis, Citespace

## Abstract

**Background:**

With the continuous development of medical imaging informatics technology, radiomics has become a new and evolving field in medical applications. Radiomics aims to be an aid to support clinical decision making by extracting quantitative features from medical images and has a very wide range of applications. The purpose of this study was to perform a bibliometric and visual analysis of scientific results and research trends in the research application of radiomics in glioma.

**Methods:**

We searched the Web of Science Core Collection (WOScc) for publications related to glioma radiomics. A bibliometric and visual analysis of online publications in this field related to countries/regions, authors, journals, references and keywords was performed using CiteSpace and R software.

**Results:**

A total of 587 relevant literature published from 2012 to September 2022 were retrieved in WOScc, and finally a total of 484 publications were obtained according to the filtering criteria, including 393 (81.20%) articles and 91 (18.80%) reviews. The number of relevant publications increases year by year. The highest number of publications was from the USA (171 articles, 35.33%) and China (170 articles, 35.12%). The research institution with the highest number of publications was Chinese Acad Sci (24), followed by Univ Penn (22) and Fudan Univ (21). WANG Y (27) had the most publications, followed by LI Y (22), and WANG J (20). Among the 555 co-cited authors, LOUIS DN (207) and KICKINGEREDER P (207) were the most cited authors. FRONTIERS IN ONCOLOGY (42) was the most published journal and NEURO-ONCOLOGY (412) was the most co-cited journal. The most frequent keywords in all publications included glioblastoma (187), survival (136), classification (131), magnetic resonance imaging (113), machine learning (100), tumor (82), and feature (79), central nervous system (66), IDH (57), and radiomics (55). Cluster analysis was performed on the basis of keyword co-occurrence, and a total of 16 clusters were formed, indicating that these directions are the current hotspots of radiomics research applications in glioma and may be the future directions of continuous development.

**Conclusion:**

In the past decade, radiomics has received much attention in the medical field and has been widely used in clinical research applications. Cooperation and communication between countries/regions need to be enhanced in future research to promote the development of radiomics in the field of medicine. In addition, the application of radiomics has improved the accuracy of pre-treatment diagnosis, efficacy prediction and prognosis assessment of glioma and helped to promote the development into precision medicine, the future still faces many challenges.

## 1 Introduction

The Central Brain Tumor Registry of the United States (CBTRUS) statistical reports that of the primary brain and other Central Nervous System (CNS) tumors diagnosed in the United States from 2014-2018, overall, meningiomas were the most common CNS tumor histology, followed by pituitary tumors and glioblastomas, with glioblastomas being the most common malignant CNS tumor (49.1%) (1). With the development of pathology and the progress of pathological detection technology, especially the improvement of the Next generation sequencing and DNA methylation profile, the genetic background and mechanism of occurrence and development of glioma are gradually clear. More and more molecular markers have been proved to play an important role in the classification, classification, grading, prognosis and treatment of gliomas. The fifth edition of the World Health Organization (WHO) Classification of Tumors of the Central Nervous System (CNS) (WHO CNS5), published in 2021, integrates the histological characteristics and molecular phenotype of tumors, puts forward a new tumor classification standard, and focuses on promoting the application of molecular diagnosis in the classification of central nervous system tumors (2). In among them, WHO CNS5 simplification of the classification of common, adult-type, diffuse gliomas. Includes only 3 types: Astrocytoma, IDH-mutant; Oligodendroglioma, IDH-mutant and 1p/19q-codeleted; and Glioblastoma, IDH-wildtype (2). The standard treatment protocol for glioma is maximal surgical resection followed by radiotherapy combined with temozolomide (TMZ) chemotherapy, and patients have a median survival of approximately 14.6 months (3). In contrast, differences in survival and treatment response of glioma are attributed to their genetic and histological characteristics, particularly isocitrate dehydrogenase (IDH) mutation status, 1p/19q co-deletion status and tumor grade (4). There is significant genetic heterogeneity within the tumor, but it needs to be assessed by molecular testing after invasive examination or surgical resection. A reproducible and non-invasive technique to predict molecular expression, therapeutic efficacy and prognostic assessment of gliomas is urgently needed.

The diagnosis of solid tumors is highly dependent on imaging, including computed tomography (CT), magnetic resonance imaging (MRI), and positron emission tomography (PET), with MRI being by far the most commonly used imaging modality for patients with brain tumors (5). However, conventional structure-based medical imaging is subjective and a qualitative assessment, and there are many shortcomings in these conventional examination methods that require the continuous development of new imaging techniques for better sensitivity and specificity, and higher temporal and spatial resolution (6). In 2012, Dutch scholar Lambin proposed the concept of radiomics based on previous work (7). Lambin concluded that “high-throughput extraction of a large number of features from medical images and transformation of imaging data into a mineable data space with high resolution through automated or semi-automated analysis methods”, medical imaging can provide a comprehensive, non-invasive and quantitative view of the spatial and temporal heterogeneity of tumors. Later, Kumar V et al. (8) expanded the definition of radiomics, which refers to the high-throughput extraction and analysis of a large number of advanced and quantitative imaging features from medical imaging images such as CT, PET or MRI. This concept was proposed and rapidly improved and refined by an increasing number of scholars in the following years. The advent of radiomics has attracted widespread interest from researchers to extract quantitative imaging features from conventional medical images and these features can also be combined with pathology and molecular biomarkers to more accurately assess the biological status of tumors and treatment response (9, 10), in addition to allowing disease stratification and potentially advancing the development of individualized treatment plans for patients (11).

In contrast to conventional imaging pictures, radiomics provides valuable quantitative information linked to biological features. Recent studies have shown that radiomics has a wide range of applications in identifying primary tumors, differential diagnosis, tumor grading, assessing gene mutation status and infiltration and heterogeneity, predicting treatment response, prognostic assessment and recurrence (12). Lohmann P et al. (13) reported the use of PET radiomics to differentiate pseudoprogression from early tumor progression in patients with glioma after chemotherapy, and the results showed that PET radiomics helps to diagnose patients with pseudoprogression with high diagnostic performance. Given its clinical significance, further studies are warranted. Yan J et al. (14) investigated quantitative radiomics based on preoperative MRI for non-invasive prediction of molecular subtypes and survival in glioma patients, and showed that MRI-based radiomics may be useful for non-invasive detection of molecular groups and prediction of survival in glioma regardless of grade. Wang J et al. (15) combined radiomic features of multisequence MRI as a pre-treatment noninvasive predictor of overall survival (OS) and chemotherapy benefit in lower-grade glioma (LGG), showed that radiomic features are independent of clinicopathological data and are a noninvasive pre-treatment predictor of survival in patients with LGG. In addition, it can predict which LGG patients will benefit from chemotherapy. Lu CF et al. (16) proposed a three-level machine learning model based on multimodal MR radiomics for the classification of glioma subtypes. The MR radiomics-based approach provides a reliable option for determining the histological and molecular subtypes of gliomas. Li G et al. (17) constructed a stable and verifiable preoperative T2-weighted MRI-based radiomic model that predicted radiomic features in the model related to immune response, especially infiltration of tumor macrophages. Preoperatively, it can stably predict the survival of glioma patients and assist in preoperative assessment of macrophage infiltration in glioma tumors. Radiomics extracts mineable data from medical images and has been widely studied and applied in clinical diseases to support clinical decision making with the aim of achieving precision medicine (18).

Radiomics has become a new and evolving field in clinical medicine, but no studies have been conducted on bibliometric methods to analysis its application in glioma. Bibliometrics uses mathematical and statistical measures to qualitatively and quantitatively evaluate the literature in the relevant field (19), and also helps researchers to quickly get a grasp of the research hotspots and trends in the field. CiteSpace is a bibliometric analysis software developed by Prof. Chaomei Chen as a tool for visual analysis of academic literature in a research field for research (20). The aim of this study is to comprehensively analysis the research application of radiomics in glioma from multiple perspectives through bibliometric tools and to fully analysis the development status and trends in this field.

## 2 Materials and methods

### 2.2 Data source

**Figure 1:**
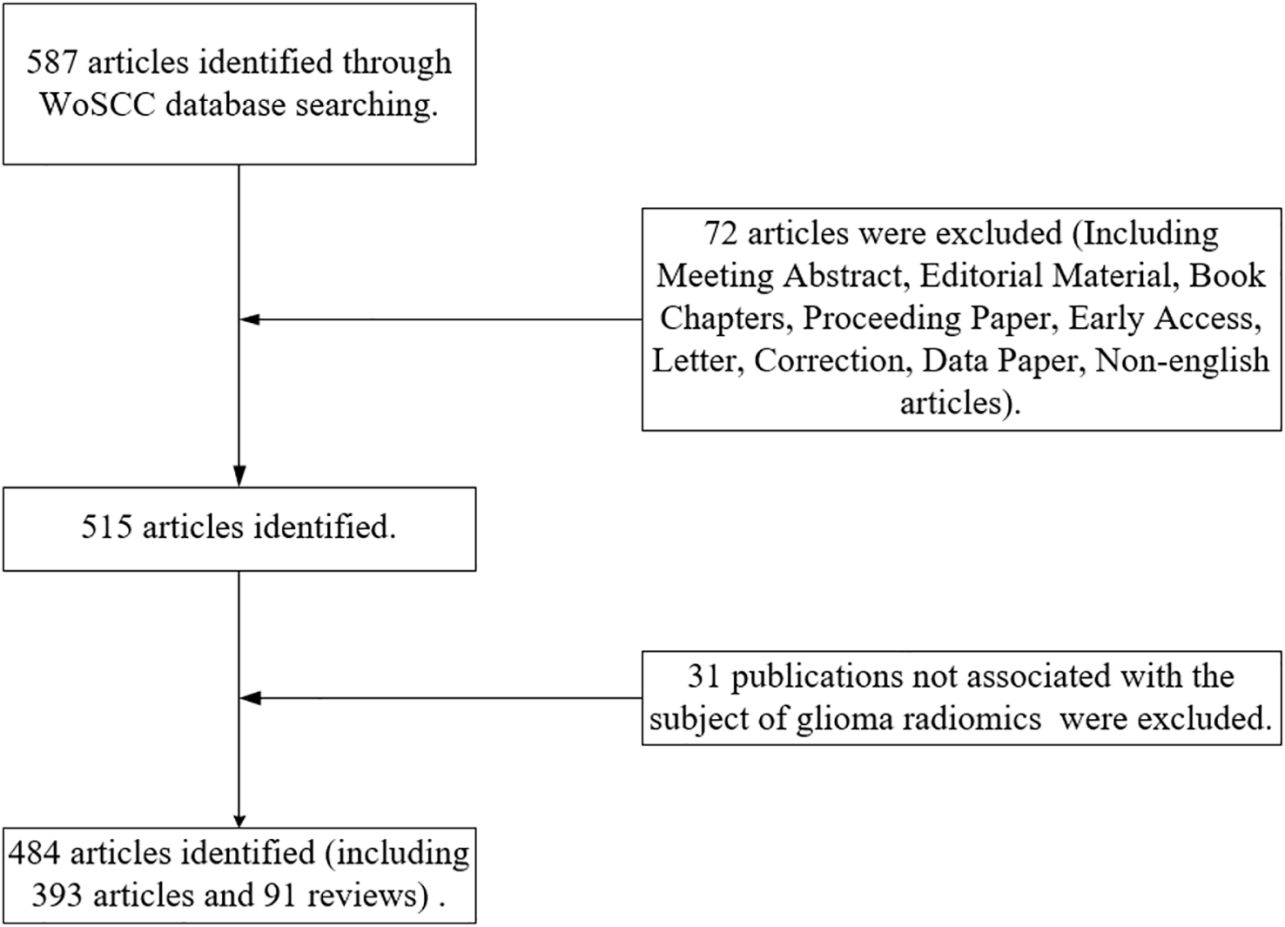
Flow chart of the literature screening process.

In September 2022, a relevant search was conducted in the Web of Science Core Collection (WOScc) database, and the time span of the search dates was set from 2012 to September 2022. The search strategy was: ((((((((((((((((TS=(glioblastoma*)) OR TS=(“glioblastoma multiform*”)) OR TS=(“malignant glioma”)) OR TS=(“brain cancer”)) OR TS=(gliosarcoma)) OR TS=(spongioblastoma)) OR TS=(astrocytoma)) OR TS=(astrocytomas)) OR TS=(“astrocytic tumors”)) OR TS=(“astrocytic glioma”)) OR TS=(“astrocyte tumors”)) OR TS=(“astrocytic glioma”)) OR TS=(oligodendroglioma*)) OR TS=(“oligodendroglial tumors”)) OR TS=(GBM)) OR TS=(LGG)) AND (((TS=(Radiomics)) OR TS=(radiogenomics)) OR TS=(“imaging omics”)). Literature inclusion criteria: (1) glioma radiomics studies were the subject; (2) the type of literature included articles and reviews; (3) the language of the literature was English. Literature exclusion criteria: (1) publications were conference abstracts, news, and case studies. (2) The study topic was not glioma. Two reviewers assessed and screened all retrieved publications, and any disagreements were resolved through discussion until consensus was reached. Flow chart of the literature screening process (**Figure 1**).

### 2.2 Analysis methods

CiteSpace software (version 6.1.3) and R software (version 4.1.3) were mainly used for bibliometric and visual analysis of publications related to the field of glioma radiomics research, and Microsoft Excel 2019 was used for data management and trend analysis of publications. CiteSpace software is a tool for visual analysis of literature developed by Prof. Chaomei Chen for analysis of indicators such as country/region, author, institution, journal, reference, and keywords (20). In addition, CiteSpace is used for keyword burst analysis and visualizes it to predict trends in the field. In the visual mapping, the node size represents the frequency of occurrence, with larger nodes representing higher frequency of occurrence. Connections between nodes represent collaboration or co-occurrence relationships. Betweenness centrality is an important parameter in CiteSpace, generally centrality ≥ 0.1 is considered as a more important node, marked by a purple circle in the visual mapping, which mainly measures the value of the node playing a bridging role in the overall network structure. The “bibliometrix” package in the R software was used for the visual analysis of the publications’ source journals.

## 3 Results

### 3.1 Publishing trend

**Figure 2:**
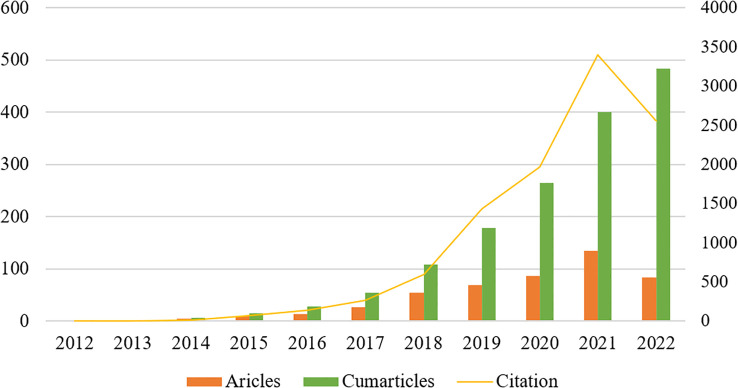
The annual number of relevant publications and the annual cumulative number of publications from 2012 to September 2022.

A total of 587 publications related to the field of glioma radiomics research from 2012 to September 2022 were searched in WOScc, and 484 were finally obtained according to the search criteria, including 393 articles (81.20%) and 91 reviews (18.80%). The number of relevant publications increased year by year, and **Figure 2** shows the trend of publications, including the annual and cumulative number of publications. The annual number of relevant publications was within 10 from 2012-2015, and the number of publications steadily increased from 2016-2020, but the annual number of publications was within 100, and the number of publications exceeded 100 in 2021. Further, we conducted a Pearson correlation analysis to test the correlation between publications and citations by Pearson correlation coefficient, and a p-value < 0.05 was considered as a significant correlation. The results showed a high positive correlation between publications and citations (r=0.97, p<0.01).

### 3.2 Country/region and institution distribution

**Figure 3:**
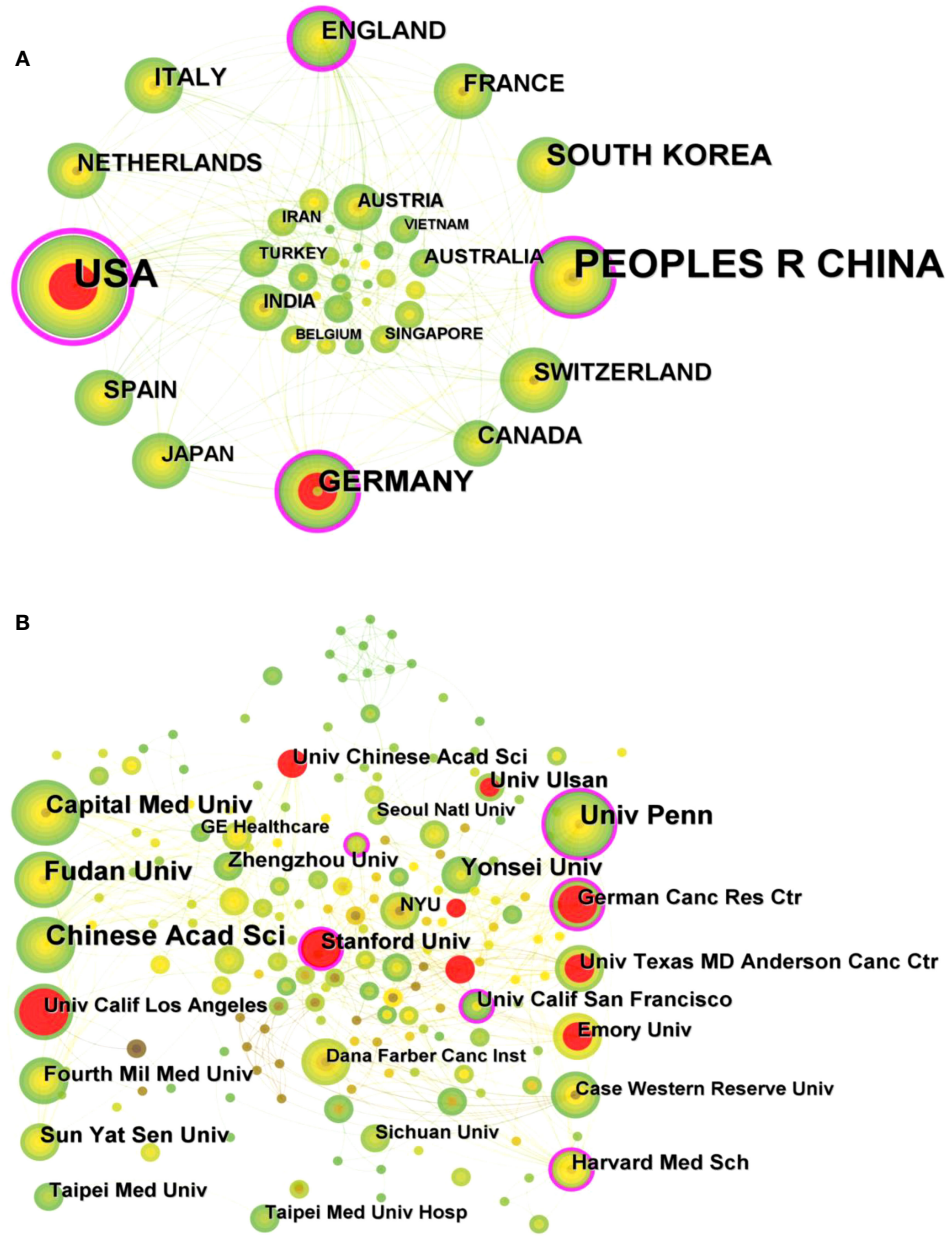
**(A)** Visual mapping of collaborative relationships between countries/regions of related publications. **(B)** Visual mapping of collaborative relationships between related publication institutions.

A total of 484 publications from 40 countries/regions and 258 institutions were obtained. The highest number of publications was from the USA (171, 35.33%) and China (170, 35.12%), followed by SOUTH KOREA (45, 9.30%) and GERMANY (41, 8.47%) (**Table 1**). **Figure 3A** shows the visual mapping of collaborative relationships between countries/regions. USA collaborates most closely with other countries/regions, followed by ENGLAND, SPAIN. The top 10 countries/regions with the highest centrality of publications are USA (0.64), followed by ENGLAND (0.24), GERMANY (0.15) and CHINA (0.13). The research institution with the most publications was Chinese Academy of Sciences (Chinese Acad Sci) (24), followed by University of Pennsylvania (Univ Penn) (22) and Fudan University (Fudan Univ) (21). Among the top 10 institutions in terms of publications, University of California, San Francisco (Univ Calif San Francisco) (0.16) had the highest centrality, followed by Stanford University(Stanford Univ)(0.13) and University of Pennsylvania(Univ Penn)(0.12) (**Table 1**). **Figure 3B** shows a visual mapping of the research institutions in CiteSpace. A node represents an institution, with larger nodes indicating more publications and higher centrality marked by purple circles.

**Table 1:** Top 10 countries/regions and institutions for related publications.

Rank	Count	Centrality	Year	Countries/regions	Count	Centrality	Year	Institution
1	171	0.64	2012	USA	24	0.07	2017	Chinese Acad Sci
2	170	0.13	2015	PEOPLES R CHINA	22	0.12	2016	Univ Penn
3	45	0.01	2017	SOUTH KOREA	21	0.1	2016	Fudan Univ
4	41	0.15	2015	GERMANY	17	0.03	2015	Capital Med Univ
5	22	0.24	2017	ENGLAND	14	0.03	2018	Yonsei Univ
6	22	0.05	2016	ITALY	12	0.02	2019	Univ Ulsan
7	20	0.03	2015	FRANCE	12	0.01	2017	Sun Yat Sen Univ
8	19	0.06	2015	NETHERLANDS	11	0.13	2014	Stanford Univ
9	18	0.02	2018	CANADA	10	0.16	2018	Univ Calif San Francisco
10	18	0	2017	SPAIN	10	0	2019	Zhengzhou Univ

### 3.3 Authors and co-cited authors

**Figure 4:**
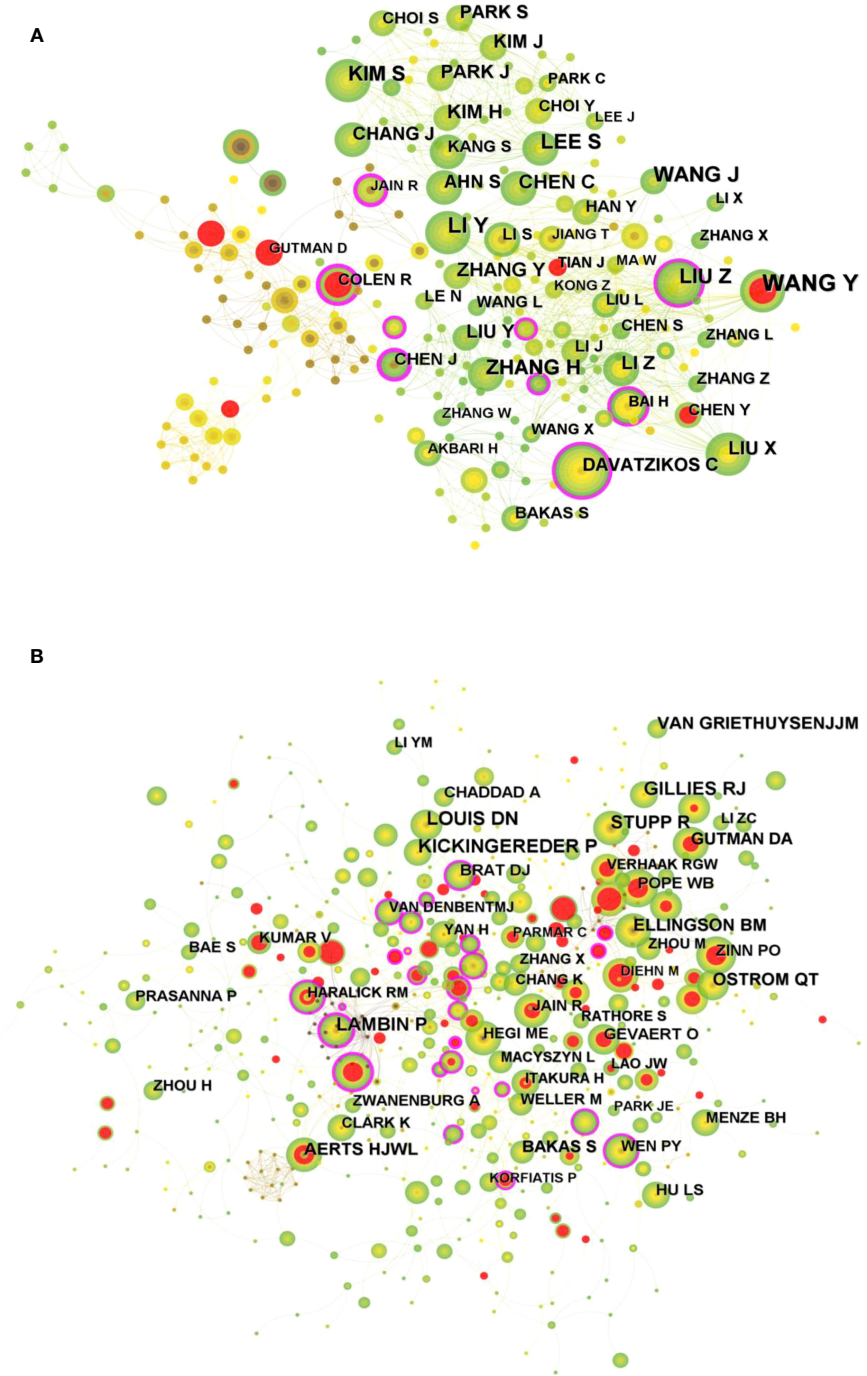
**(A)** Visual mapping of authors of related publications in CiteSpace. **(B)** Visual mapping of co-cited authors of related publications in CiteSpace.

A total of 354 researchers were involved in the publication of relevant literature. WANG Y (27) had the highest number of publications, followed by LI Y (22), WANG J (20), and among the top 10 authors LIU Z (0.1) had the highest centrality (**Table 2**). **Figure 4A** shows the visual mapping of author collaboration network, each circle represents an author, the larger the circle the more publications, the line between the circles represents the connection between authors, and the thicker the line the closer the collaboration. In 1973, Small, an American intelligence scientist, first introduced the concept of co-citation as a method to measure the degree of relationship between documents (21). In 1981, White and Griffith extended the co-citation of literature to the author level and developed the method of author co-citation analysis (ACA) (22). In addition, there are journal co-citations. Co-cited authors are two or more authors who are cited in one or more articles at the same time, and these two or more authors form a co-citation relationship. Among the 555 co-cited authors, LOUIS DN (207) and KICKINGEREDER P (207) were the most co-cited authors, followed by LAMBIN P (171), GILLIES RJ (165). Among the top 10 co-cited authors LAMBIN (0.23) had the highest centrality (**Table 2**). **Figure 4B** shows a visual mapping between the co-cited authors.

**Table 2:** Top 10 authors and co-cited authors in related publications.

Rank	Count	Centrality	Authors	Rank	Count	Centrality	Co-Cited Authours
1	27	0.05	WANG Y	1	207	0	LOUIS DN
2	22	0.05	LI Y	2	207	0.05	KICKINGEREDER P
3	20	0.03	WANG J	3	171	0.23	LAMBIN P
4	18	0.1	LIU Z	4	165	0.01	GILLIES RJ
5	18	0.05	LEE S	5	139	0.02	STUPP R
6	18	0.05	KIM S	6	126	0.08	AERTS HJWL
7	18	0.03	ZHANG H	7	125	0	OSTROM QT
8	16	0	KIM H	8	119	0	ELLINGSON BM
9	15	0.01	CHEN C	9	116	0	VAN GRIETHUYSENJJM
10	15	0.07	ZHANG Y	10	99	0.02	GUTMAN DA

### 3.4 Journals and co-cited journals

**Figure 5:**
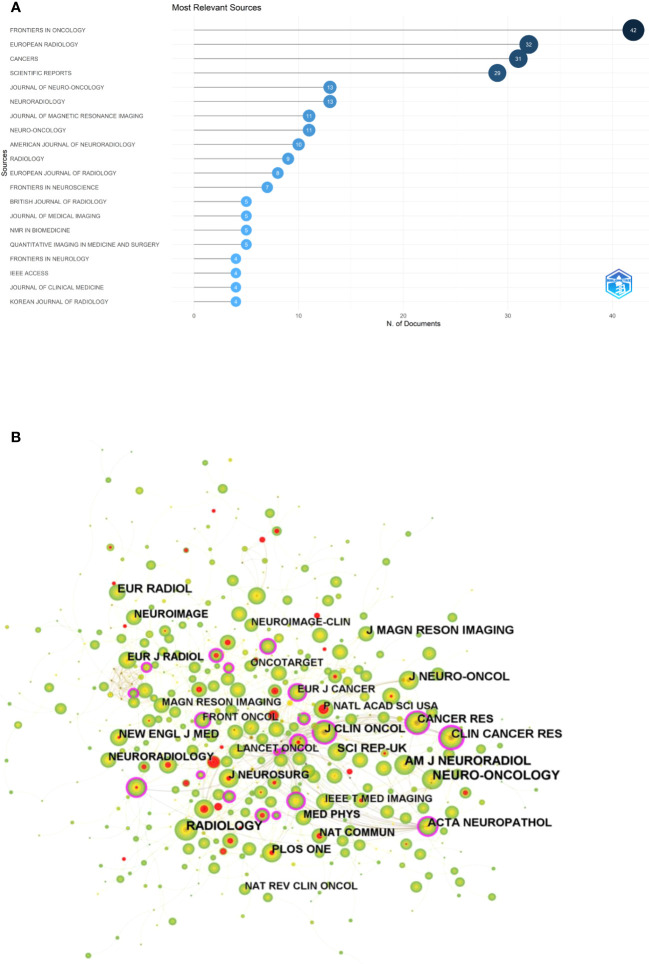
**(A)** Visual mapping of the top 20 source journals for related publications. **(B)** Visual mapping of co-cited journals of related publications.

The “bibliometrix” package in R software (version 4.1.3) was used to visually analysis the source journals of the relevant publications. FRONTIERS IN ONCOLOGY (42) was the most published journal, followed by EUROPEAN RADIOLOGY (32) and CANCERS (31). **Figure 5A** shows a visual mapping of the top 20 academic journals in terms of number of articles published. Among the top 10 academic journals, the highest impact factor is RADIOLOGY (29.146), followed by NEURO-ONCOLOGY (13.029) (**Table S1**). The most cited of the 516 co-cited journals was NEURO-ONCOLOGY (412), followed by RADIOLOGY (402) and AM J NEURORADIOL (345) (**Table 3**). Among the top 10 co-cited journals, NEW ENGL J MED (176.709) had the highest impact factor and CLIN CANCER RES (0.27) had the highest centrality, indicating the high status of these journals in this research area. **Figure 5B** shows the visual mapping of co-cited journals, with larger circles representing higher frequency of co-citations and purple circles indicating higher centrality.

**Table 3:** Top 10 co-cited journals for related publications.

Rank	Count	Centrality	Cited Journals	IF 2022	Quartile in category
1	412	0.05	NEURO-ONCOLOGY	13.029	Q1
2	402	0	RADIOLOGY	29.146	Q1
3	345	0.04	AM J NEURORADIOL	4.966	Q2
4	305	0	EUR RADIOL	7.034	Q1
5	302	0.01	SCI REP-UK	4.996	Q2
6	289	0.01	J NEURO-ONCOL	4.506	Q2
7	252	0.27	CLIN CANCER RES	13.801	Q1
8	246	0.04	J MAGN RESON IMAGING	5.119	Q1
9	238	0	NEW ENGL J MED	176.709	Q1
10	237	0.01	PLOS ONE	3.752	Q2

Chen C et al. (23) introduced a new visual analysis method, dual-map overlay, which is used to analyze, compare and contrast the characteristics of publications portfolios. The new method introduces a novel dual-map thematic overlay design on global science. Each publication portfolio can be superimposed as a one-layer of dual-map overlays over 2 related, but different global science maps: one for the citing journal and the other for the cited journal. The color paths in **Figure S1** shows the citation relationships, where the three green paths, respectively, indicate that literature published in dentistry, dermatology, surgery journals is frequently cited by Molecular, Biology, Genetics. Literature from Medicine, Biology, clinical journals is often cited in health, nursing, medicine journals. Literature from neurology, sports, ophthalmology journals is often cited in psychology, education, social journals.

### 3.5 Co-cited references and references burst

**Figure 6:**
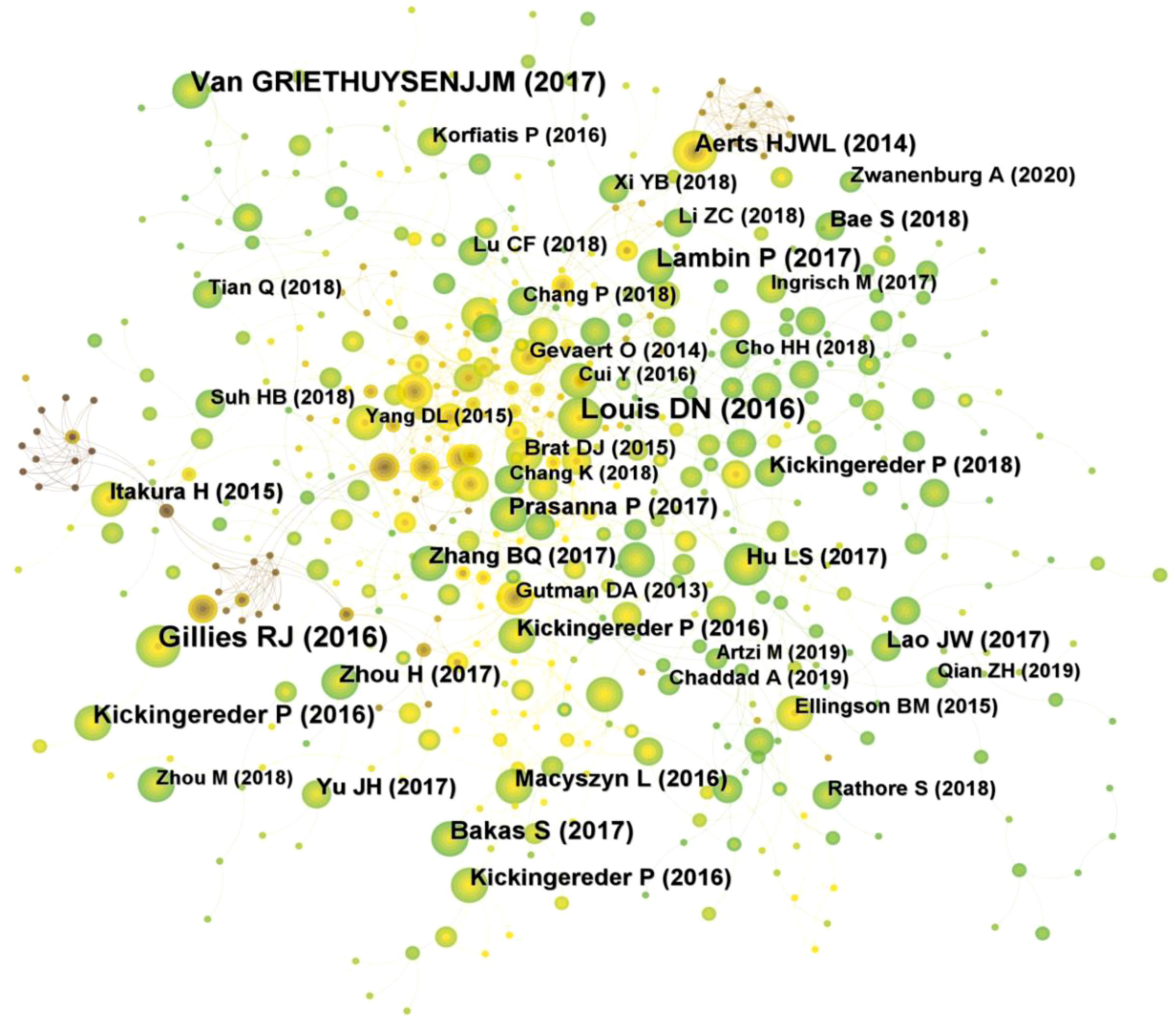
Visual mapping of references to related publications.

Of the 558 co-cited references retrieved, the top 10 most frequently cited references are listed in **Table 4**. **Figure 6** shows a visual mapping of the co-cited references. Gillies RJ et al. (24) reported the most frequently cited article “Radiomics: Images Are More than Pictures, They Are Data”. Describes the process of radiomics, its challenges, and its potential power to facilitate better clinical decision making, particularly in the care of cancer patients. Second, van Griethuysen JJM et al. (25) report on the “Computational Radiomics System to Decode the Radiographic Phenotype”, which reports on their team’s development of PyRadiomics, a flexible open-source platform capable of extracting a large number of engineering features from medical images. PyRadiomics is implemented in Python and can be used standalone or with 3D Slicer. And discusses the workflow and architecture of PyRadiomics and demonstrates its application to lung lesions characterization. In addition, according to the titles of the top 10 co-cited references, it is possible to understand that their topics are mainly about the prediction of survival in glioma patients by radiomics, and the study of molecular subtypes.

**Table 4:** Top 10 co-cited references in related publications.

Rank	Count	Centrality	Cited Reference
1	136	0.01	Radiomics: Images Are More than Pictures, They Are Data
2	136	0.01	The 2016 World Health Organization Classification of Tumors of the Central Nervous System: a summary
3	115	0.01	Computational Radiomics System to Decode the Radiographic Phenotype
4	90	0.03	Radiomics: the bridge between medical imaging and personalized medicine
5	75	0.01	Radiomic Profiling of Glioblastoma: Identifying an Imaging Predictor of Patient Survival with Improved Performance over Established Clinical and Radiologic Risk Models
6	71	0	Data Descriptor: Advancing The Cancer Genome Atlas glioma MRI collections with expert segmentation labels and radiomic features
7	62	0.09	Decoding tumor phenotype by noninvasive imaging using a quantitative radiomics approach
8	53	0.01	Radiomic features from the peritumoral brain parenchyma on treatment- naïve multi-parametric MR imaging predict long versus short-term survival in glioblastoma multiforme: Preliminary findings
9	52	0.05	Radiogenomics of Glioblastoma: Machine Learning-based Classification of Molecular Characteristics by Using Multiparametric and Multiregional MR Imaging Features
10	52	0.03	MRI features predict survival and molecular markers in diffuse lower-grade gliomas

Reference burst analysis is beneficial for researchers to understand the literature in a field that has received focused attention during a certain period of time. Based on the strongest citation bursts, **Figure S2** shows that the first citation burst started in 2011, with the top 25 references having a citation burst intensity of 4.64-19.18. Among them, the strongest and longest lasting citation was published by Aerts HJ et al. (26) in the journal “Nat Commun”, entitled “Decoding tumor phenotype by noninvasive imaging using a quantitative radiomics approach”, reported the prognostic power of a large number of radiomic features, many of which had not previously been identified as significant before, in an independent dataset of patients with lung and head and neck cancers. Radiogenomics analysis revealed that prognostic radiomic features capturing intra-tumor heterogeneity were associated with underlying gene expression patterns. These findings may have clinical implications as imaging is routinely used in clinical practice, providing an unprecedented opportunity for low-cost improved decision support for cancer treatment.

### 3.6 Keyword visual analysis

**Figure 7:**
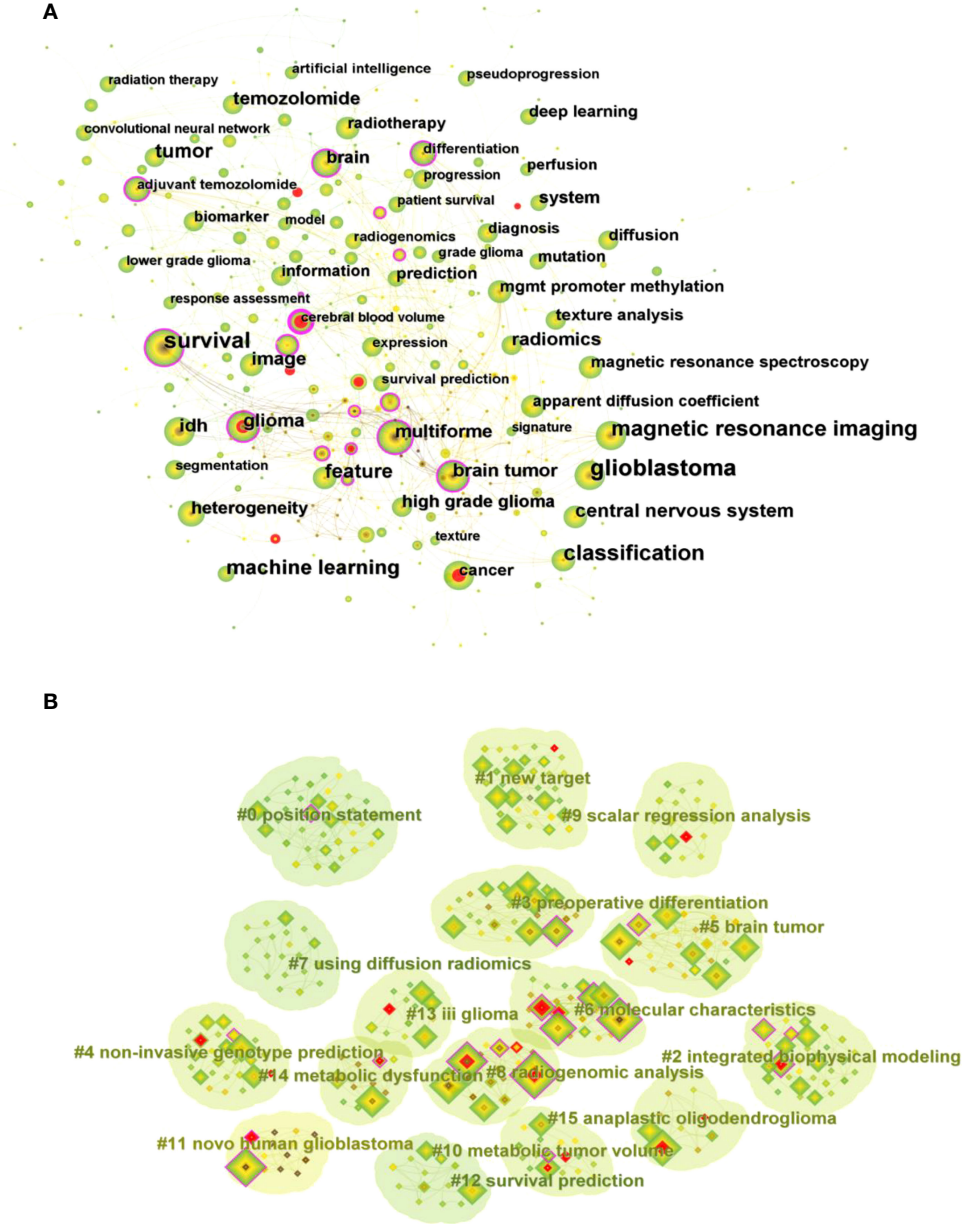
**(A)** Visual mapping of keyword co-occurrence for related publications. **(B)** Visual mapping of keyword clustering for related publications.

Keywords are words or phrases selected to reflect the concept of the subject of a paper for the purpose of literature indexing and searching. The analysis of keywords enables to summarize the research themes in a specific field and explore hot spots and research directions. The top 10 keywords that appear frequently in the studies related to glioma imaging histology displayed in **Table S2** include glioblastoma (187), survival (136), classification (131), magnetic resonance imaging (113), machine learning (100), tumor (82), feature (79), central nervous system (66), IDH (57), and radiomics (55), indicating that these areas are the current research hotspots in glioma radiomics. In the keyword co-occurrence virtual mapping (**Figure 7A**), there are 344 nodes and 2211 connected lines. Each node corresponds to a keyword, and the larger the node the more frequently the keyword appears. The number of connecting lines between nodes and the distance between nodes reflect the closeness of the keywords.

The clustering analysis of keywords based on keyword co-occurrence analysis can reflect the hot directions of this research area. **Figure 7B** shows the visual mapping of keyword clustering, which mainly includes high-grade glioma treatment response monitoring, glioblastoma patient survival prediction, programmed death-ligand, preoperative differentiation, non-invasive genotype prediction, brain tumor, molecular characteristics, machine learning model development, illuminating radiogenomic characteristics, scalar regression analysis, using radiomics, anatomic localization, artificial intelligence, predicting early recurrence, genomic mapping, regional genetic heterogeneity 16 clusters.

## 4 Discussion

### 4.1 General information

Publication volume is a more intuitive reflection of the research fervor and development rate of this research field within a certain period of time, which is important for analysis of research dynamics and prediction of development trends. **Figure 2** shows that the annual and cumulative number of publications related to glioma radiomics are both on the rise. 2012-2015, the annual number of related publications was within 10, and the number of publications steadily increased from 2016 to 2020, but the annual number of publications was within 100, and the number of publications exceeded 100 in 2021, indicating that this field is receiving more and more attention from researchers and the research intensity is keeps rising.

The volume and centrality of publications by country/region and institution provides an objective reflection of the level of scientific research in the relevant research area and the value of acting as a bridge in the entire network structure. The highest number of publications is in USA (171, 35.33%) and China (170, 35.12%), with USA working most closely with other countries. In addition, the country/region with the highest centrality among the top 10 countries/regions in terms of publication volume is USA (0.64), followed by ENGLAND (0.24), GERMANY (0.15) and China (0.13), which shows that USA has contributed greatly to the scientific development in this field. The research institution with the highest number of publications is Chinese Acad Sci (24), and among the top 10 institutions in terms of publications, Univ Calif San Francisco (0.16) has the highest centrality. In the future, exchange and cooperation between countries/regions need to be strengthened to promote the development of this research area.

A total of 354 researchers were involved in the publication of related literature. WANG Y (27) had the most publications, followed by LI Y (22), WANG J (20), and among the top 10 authors LIU Z (0.1) had the highest centrality. Wang Y et al. (27) build a radiomic prediction model of LGG-related epilepsy types based on MRI data. The results showed that MRI-based radiomic analysis could predict the type of LGG-related epilepsy and thus provide individualized treatment for patients with LGG-related epilepsy. Among the 555 co-cited authors, LOUIS DN (207) and KICKINGEREDER P (207) were the most cited authors. Among the top 10 co-cited authors LAMBIN (0.23) had the highest centrality. Louis DN et al. reported “2016 World Health Organization classification of central nervous system tumors: summary”, proposed a concept for the structure of CNS tumor diagnosis in the molecular era. Building on the 2016 update of the fourth edition and the work of the CNS Consortium for Molecular and Practical Approaches to Tumor Taxonomy, the 2021 fifth edition introduces significant changes that advance the role of molecular diagnostics in the classification of CNS tumors (2). It is evident that Louis DN and his colleagues have made a great contribution to the continuous updating of the tumor classification of CNS. Kickingereder P et al. (28) developed a framework that relies on artificial neural networks (ANNs) for fully automated quantitative analysis of MRI in neuro-oncology to overcome the inherent limitations of manual assessment of tumor burden. The discovery of ANN enables objective and automated assessment of tumor response in neuro-oncology at high throughput and could eventually serve as a blueprint for the application of ANN in radiology to improve clinical decision making. Future research should focus on prospective validation in clinical trials, as well as application to automated high-throughput imaging biomarker discovery and extension to other diseases. In addition, Kickingereder P et al. (29) explored the correlation between multiparametric, multiregional MRI features and key molecular features in patients with newly diagnosed glioblastoma. Found associations between established MRI features and molecular features, although their strength is not sufficient to generate machine learning classification models for reliable and clinically meaningful prediction of molecular features in patients with glioblastoma. In addition, the role of integrating radiomics into a multi-layered decision-making framework with key molecular and clinical features to improve disease stratification and potentially advance personalized treatment of glioblastoma patients is emphasized (11).

FRONTIERS IN ONCOLOGY (42) was the most published journal, followed by EUROPEAN RADIOLOGY (32) and CANCERS (31). Among the top ten academic journals, RADIOLOGY (29.146) has the highest impact factor, followed by NEURO-ONCOLOGY (13.029). The impact factor of journals is widely accepted and recognized internationally, and is an important indicator for evaluating the academic influence of journals. The most cited of the 516 co-cited journals was NEURO-ONCOLOGY (412), followed by RADIOLOGY (402) and AM J NEURORADIOL (345), and among the top 10 co-cited journals, NEW ENGL J MED (176.709) had the highest impact factor and CLIN CANCER RES (0.27) had the highest centrality. This indicates that the above journals have high influence and status in this research area.

The analysis of co-cited references can reveal the research themes clustered in the field. Information on the top 10 co-cited references was mined, which can be considered as the classic literature in the field. Based on the top 10 co-cited references it can be understood that the research themes in this field are mainly about the prediction of survival of glioma patients by radiomics, and molecular subtypes. The reference with the greatest burst intensity and longest duration was reported by Aerts HJ et al. (26), in an independent dataset of patients with lung and head and neck cancer, a large number of radiogenomic features with prognostic power, many of which had not previously been identified as significant.

Generally, keywords in the literature are the core summary of research content. Most scholars use keywords in the literature of a specific field to analyze the research themes and hotspots in different periods and reveal the trajectory of changes in the research content and research focus of a specific field. We listed the top ten keywords in this research field, and performed keyword clustering analysis based on keyword co-occurrence, which finally formed 16 clusters to identify the current research hotspots and possible future development trends of radiomics in glioma. The main contents are as follows:

#### 4.1.1 Overview of radiomics

Radiomics, an emerging field, rapidly extracts numerous quantitative features from tomographic images such as CT, MR, or PET images through high-throughput calculations. The process of converting digital medical images into mineable high-dimensional data, called radiomics, motivates the reflection of information about underlying pathophysiology through medical images, and these relationships can be revealed by quantitative image analysis. This multi-step process involves image acquisition and reconstruction, image preprocessing, identification of regions of interest (ROI), feature extraction and quantization, feature filtering, and predictive model building (24). In image acquisition, the quality of MR images obtained with different instruments and imaging parameters varies widely. Such imaging quality variation will have a significant impact on the radiomics analysis. The main differences in MR images include mode mismatch (M), intensity distribution differences (I) and layer spacing differences (L), which are referred to as MIL differences. Hu Z et al. (30) proposed a MIL normalization system to reconstruct inhomogeneous MR images into high-quality data with complete modes, uniform intensity distribution and consistent layer spacing. The MIL normalization system provides high-quality standardized data, which is a prerequisite for accurate radiomics analysis. Typical image preprocessing for radiomics analysis includes, but is not limited to, intensity normalization, spatial smoothing, spatial resampling, noise reduction, and correction of MRI field inhomogeneities (8, 31). Feature-based radiomics utilizes a set of mathematically predefined features that are typically extracted from a segmented ROI or volume-of-interest (VOI), and image segmentation can be achieved by manual, semi-automatic or fully automatic methods (32, 33). After feature extraction, a subset of relevant features is determined by feature selection algorithms to avoid overfitting and to generate robust and generalizable predictive models. Parmar C et al. (34) evaluated 14 feature selection methods and 12 classification methods for predictive performance and stability to data perturbations. The results showed that the Wilcxon-test based feature selection method WLCX produced the highest prediction performance in most classifiers. Interestingly, WLCX is a simple univariate rank-based method that does not consider the redundancy of selected features during feature ranking. Most feature selection methods provide the highest predictive performance when used with random forest (RF) classifiers. Commonly used feature selection algorithms include the minimum redundancy maximum relevance (mRMR) algorithm (35, 36) and the sequential feature selection methods (37). After feature selection, a mathematical model for the prediction of a known, underlying ground truth. The most popular algorithms in radiomics are linear and logistic regression, decision trees (e.g., random forests), support vector machines, neural networks, and the Cox proportional hazards model in case of censored survival data (38). Since high variation in medical imaging parameters affects the robustness of radiomic features and thus the performance of the predictive models built on them. Reiazi R et al. (39) evaluated the impact of imaging parameters on the robustness of radiomic features. Insights are also provided on the validity and variability of different methods that have been applied to investigate the robustness of radiological features. Radiomics research still faces many problems in clinical practice, with inconsistencies in imaging equipment, acquisition parameters, and image preprocessing methods and methods of study used, resulting in widely variable results and poor reproducibility. To ensure the stability and generalizability of the study results, standardization of radiomics study methods is necessary in future studies, which will also be the focus of future research.

#### 4.1.2 Application of radiomics in glioma

##### 4.1.2.1 Radiomics applied to predict the molecular subtypes of glioma

The criteria for glioma classification in CNS was introduced by WHO in 2021. This criteria for glioma classification requires the integration of histology with genomics (2). In contrast, the current clinical gold standard for detecting chromosomal mutations is still invasive and poses a hidden risk to patients. Pei L et al. (40) proposed a new approach to glioma analysis that, for the first time, combines cellular features derived from digital analysis of brain histopathology images with molecular features that follow the latest WHO criteria. This work shows for the first time in the literature the promise of cellular quantification to predict brain tumor grading in LGGs with IDH mutations. Yu J et al. (41) explored a noninvasive approach to reveal IDH1 status by a quantitative radiomic approach in grade II gliomas, and the result showed that radiomics is a potentially useful method to estimate IDH1 mutation status noninvasively using conventional T2-Flair MRI images. Tan Y et al. (42) develop and validate a radiomic nomogram for preoperative prediction of IDH genotype in astrocytoma. The results showed that the radiomic signature was built by six selected radiomic features, yielding area under roc curve (AUC) values of 0.901 and 0.888 in the training and validation cohorts. Li Y et al. (43) develop a radiomics pipeline based on the clinical MRI scans to non-invasively predict glioma subtypes, defined based on tumor grade, IDH mutation status and 1p/19q codeletion status. Sun C et al. (44) constructed a joint machine learning-based model to predict molecular subtypes of LGG, and the results showed that the joint machine learning algorithm can provide a non-invasive method to predict preoperative molecular subtypes of LGG with good predictive performance. Wei J et al. (45) based MRI radiomics to predict MGMT methylation status, the results showed that fusion radiomics features exhibited the highest ability in predicting MGMT promoter methylation, with AUC of 0.925 in the training cohort and 0.902 in the validation cohort. Guo J et al. (46) explored the combination of multiparametric MRI-based radiomics with selected blood inflammatory markers was effective in predicting grade and proliferation in glioma patients. Radiomic features obtained from medical images have been used as a new method for non-invasive diagnosis and clinical decision making, showing clear advantages in the non-invasive histopathological and molecular diagnosis of gliomas. In contrast, the optimal combination of multiparametric MRI and machine learning techniques has not yet been determined, and the performance of combining other clinical bioindicators in predicting molecular mutation status has not yet been fully evaluated, which will be the direction and focus of future research.

##### 4.1.2.2 Radiomics applied to survival prediction in glioma

Due to the site specificity of glioma growth, heterogeneity of tumor cells and drug resistance, this still leaves glioma patients with a poor prognosis. Researchers are interested in more accurately predicting the survival rate of glioma patients, which could lead to better individualized treatment plans. Wang J et al. (15) developed and validated a radiomic signature for survival and chemotherapy efficacy in LGG patients. The results showed that combining radiomic features of combined contrast-enhanced axial T-1 weighted (CE-T1-w) and fluid-attenuated inversion recovery (LAIR) sequences with clinicopathologic nomograms was superior to clinicopathologic nomograms in predicting OS in LGG. Choi Y et al. (47) reported that multiparametric MR-based radiomics combined with conventional clinical and genetic prognostic models improved the prognostic value of OS and progression-free survival (PFS) in patients with glioblastoma. Shaheen A et al. (48) evaluated the efficacy of four multi-regional radiomics models for OS classification and quantified the robustness of predictions to automatic segmentation of brain tumor volume changes. Concluded that while STAPLE-fusion can reduce segmentation errors, it is also not a solution for learning accurate and robust radiomics models. Xu C et al. (49) used the integration of state-of-the-art convolutional neural networks (CNN) and radiomics to stratify glioma grade and predict survival of LGG patients. This proposed integrated approach can be applied noninvasively and effectively for the prediction of glioma grade and survival. Bae S et al. (50) investigated MRI radiomic features combined with clinical and genetic information. The results showed that adding radiomic models to clinical and genetic profiles improved survival prediction compared to models that included clinical and genetic profiles alone. Park JE et al. (51) developed and validated a multiparametric MR radiomics model and demonstrated that incorporating diffusion- and perfusion-weighted MR imaging into an MR radiomics model improves prognostication in glioblastoma patients with better performance over that achievable with clinical predictors alone. In order to achieve more accurate predictive efficacy, many researchers have proposed the development of hybrid prediction models, which shows that the combination of radiomic features with clinical and genetic factors will be a research trend and hot spot for survival prediction in glioma patients in the future.

##### 4.1.2.3 Radiomics applied to the differential diagnosis of glioma

Reliable assessment of tumor diagnosis by radiomics prior to treatment can help guide treatment selection and reduce the incidence of adverse events. Suh HB et al. (52) machine learning algorithms based on MRI radiomics distinguished primary central nervous system lymphoma (PCNSL) from non-necrotizing atypical glioblastoma and showed excellent predictive performance. Bathla G et al. (53) compared the diagnostic performance of several radiomics-based models in distinguishing glioblastoma from PCNSL. The predictive performance using both individual and combined sequences, which was fairly stable across multiple best performing models (AUC: 0.961-0.977), but did display considerable variation between the best and worst performing models. Revealed that the predictive accuracy of radiomics can differ significantly based on the model and feature selection methods and the combination of sequences used.

Currently, there is no reliable diagnostic test to distinguish between pseudoprogression and early tumor progression. Lohmann P et al. (13) reported that PET radiomics helped to diagnose patients with pseudoprogressive gliomas. Müller M et al. (54) developed a PET-based radiomic classifier that showed high accuracy in differentiating treatment-related changes (TRC) from tumor progression (TP) in gliomas. Distinguishing between glioblastoma and isolated brain metastases may be challenging due to the similar appearance of both on MRI. Su CQ et al. (55) reported a study using radiomic analysis to differentiate between glioblastoma and solitary brain metastases, the results showed that in the training and validation cohorts, the radiomic features yielded AUC values of 0.82 and 0.81. Ortiz-Ramón R et al. (56) evaluated the potential of 2D texture features extracted from MR images in differentiating brain metastases (BM) from glioblastomas following a radiomics approach. Results showed that the radiomics is able to discriminate between glioblastoma and BM with high accuracy using a set of 2D texture features, thus helping to diagnose brain lesions in a rapid and non-invasive way. Since there is no uniform model to obtain the best performance for each specific dataset, it is necessary to try different combinatorial approaches and combining different advanced imaging protocols is a future research direction.

##### 4.1.2.4 Radiomics applied to individualized treatment of glioma

Radiomics has made tremendous developments in the last decade, serving as a bridge between imaging and precision medicine (57). The application of radiomics research reflects the quest for precision medicine, and the availability of robust and validated biomarkers is essential to drive precision medicine forward. Fathi Kazerooni A et al. (58) described the prospects of radiomics and radiogenomics for personalized treatment of glioma patients from the perspectives of neuro-oncology, neuropathology and computational. Concluded that radiomics introduces new solutions to the current clinical challenges of glioma treatment and provides promising evidence for personalized diagnosis and treatment. Carles M et al. (59) evaluated a prognostic model based on PET radiomic signature that facilitates prognostic assessment and selection of patients with recurrent glioblastoma who benefit from re-radiotherapy, and further analysis in a larger prospective validation cohort is warranted and planned.

Noninvasive early prediction and delineation of recurrence can aid in targeted treatment, which has the potential to delay recurrence. Chougule T et al. (60) reported a study on understanding longitudinal radiomic changes from preoperative MRI to glioma recurrence and subsequently using machine learning to predict recurrence areas 143 ± 42 days before recurrence. The findings showed a step forward in the prediction of glioblastoma recurrence by phenotypic changes in radiomics, which have the potential to serve as MRI-based biomarkers for tailoring customized therapeutic interventions. Kickingereder P et al. (11) reported that integrating radiomics with key molecular and clinical features into a multilevel decision framework could improve disease stratification and potentially advance personalized therapy for patients with glioblastoma. Classifiers based on various combinations of MRI sequences, genetic information, and clinical data can predict tumor diagnosis, overall survival, and treatment response with reasonable accuracy and non-invasively. Radiomics has the potential to transform the scope of glioma management through personalized medicine, but the application in glioma is still in its infancy and has not yet been translated into clinical decision making (61). Larger sample sizes, standardized image acquisition and data extraction techniques will be needed in the future to develop machine learning models that can be effectively translated into clinical practice.

A large literature showed that radiomics has been extensively studied in glioma and has yielded promising results. However, large-scale multicenter validation of existing exploratory radiomics studies is still lacking, and the vast majority of validation cohorts are still derived from retrospective data from single independent units. In addition, correlations between radiomics and clinical features have been extensively studied, but less so with other data types such as genomics, transcriptomics, proteomics, and metabolomics. And the next milestone in radiomics will undoubtedly be the establishment of models for clinical decision support. However, achieving this goal will require the development of globally accepted standards as well as the establishment of a robust and comprehensive common database, which in turn will require the participation of different medical centers from around the world to provide data. These challenges and directions will also be the focus and trend of the field in future research.

## 5 Limitations

1) The data were obtained from WOScc only. 2) We collected relevant literature from 2012-2022, while the literature in WOScc is continuously updated. 3) Reviewers removed irrelevant literature from the study manually which may lead to selection bias.

## 6 Conclusion

We conducted a comprehensive analyze of publications related to glioma radiomics using bibliometric tools to reveal the bibliometric features of the field. A synthesis of relevant publications identifies the current state of research and research hotspots in the field. Although radiomics is a rapidly expanding field, the application in glioma is still at the stage of clinical exploration and there are many obstacles to overcome in the future.

## Author contributions

CC conceived the study and conducted the literature searched and prepared the figures. XD prepared the tables. CC performed the manuscript writing. XD, YL, HL, ZL, ZG revised the manuscript. JQ supervised and checked the manuscript. All authors contributed to the manuscript and approved the submission.
