# Supplementary material for: Research on application of radiomics in glioma: a bibliometric and visual analysis
Source: Front Oncol. 2023 Sep 12;13:1083080. doi: 10.3389/fonc.2023.1083080 (PMC10523166; doi:10.3389/fonc.2023.1083080)
Supplement: Supplementary file 1 [file DataSheet_1.pdf]

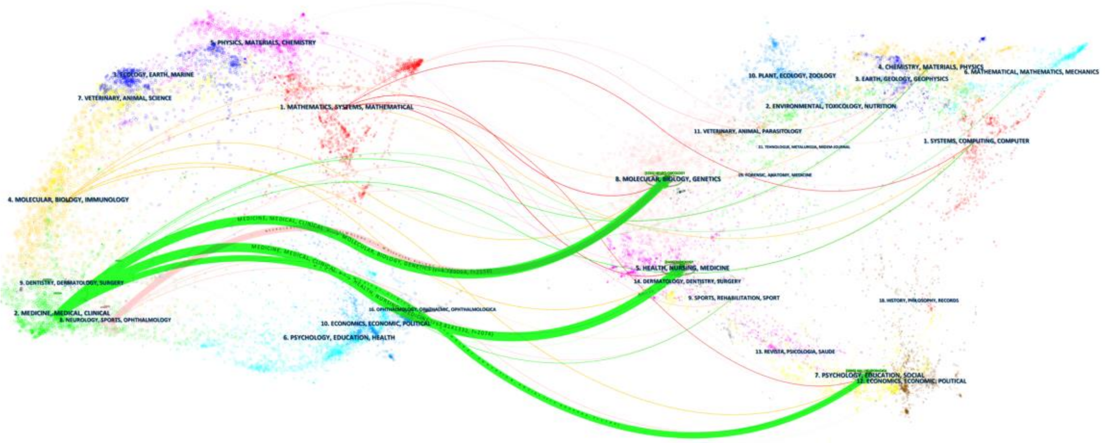

Figure S1. The dual-map overlay of journals of related publications.

Top 25 References with the Strongest Citation Bursts

References	Year	Strength	Begin	End	2012 - 2022
Zinn PO, 2011, PLOS ONE, V6, P0, DOI 10.1371/journal.pone.0025451, DOI	2011	9.5	**2013**	2016	
Zinn PO, 2012, PLOS ONE, V7, P0, DOI 10.1371/journal.pone.0041522, DOI	2012	5.06	**2013**	2016	
Aerts HJWL, 2014, NAT COMMUN, V5, P0, DOI 10.1038/ncomms5006, DOI	2014	19.18	**2014**	2019	
Gutman DA, 2013, RADIOLOGY, V267, P560, DOI 10.1148/radiol.13120118, DOI	2013	18.03	**2014**	2018	
Jain R, 2013, RADIOLOGY, V267, P212, DOI 10.1148/radiol.12120846, DOI	2013	7.35	**2014**	2018	
Carrillo JA, 2012, AM J NEURORADIOL, V33, P1349, DOI 10.3174/ajnr.A2950, DOI	2012	5.78	**2014**	2017	
Gevaert O, 2014, RADIOLOGY, V273, P168, DOI 10.1148/radiol.14131731, DOI	2014	12.74	**2015**	2018	
Naeini KM, 2013, NEURO-ONCOLOGY, V15, P626, DOI 10.1093/neuonc/not008, DOI	2013	7.95	**2015**	2018	
Lambin P, 2012, EUR J CANCER, V48, P441, DOI 10.1016/j.ejca.2011.11.036, DOI	2012	7.04	**2015**	2017	
Jamshidi N, 2014, RADIOLOGY, V270, P212, DOI 10.1148/radiol.13130078, DOI	2014	6.8	**2015**	2018	
Ellingson BM, 2013, AM J NEURORADIOL, V34, P533, DOI 10.3174/ajnr.A3253, DOI	2013	6.07	**2015**	2018	
Mazurowski MA, 2013, NEURO-ONCOLOGY, V15, P1389, DOI 10.1093/neuonc/nos335, DOI	2013	4.67	**2015**	2018	
Clark K, 2013, J DIGIT IMAGING, V26, P1045, DOI 10.1007/s10278-013-9622-7, DOI	2013	4.67	**2015**	2018	
Itakura H, 2015, SCI TRANSL MED, V7, P0, DOI 10.1126/scitranslmed.aaa7582, DOI	2015	8.08	**2016**	2018	
Jain R, 2014, RADIOLOGY, V272, P484, DOI 10.1148/radiol.14131691, DOI	2014	6.72	**2016**	2019	
Sottoriva A, 2013, P NATL ACAD SCI USA, V110, P4009, DOI 10.1073/pnas.1219747110, DOI	2013	6.36	**2016**	2018	
Brat DJ, 2015, NEW ENGL J MED, V372, P2481, DOI 10.1056/NEJMoa1402121, DOI	2015	6.2	**2016**	2020	
Yang DL, 2015, MED PHYS, V42, P6725, DOI 10.1118/1.4934373, DOI	2015	5.62	**2016**	2019	
Parmar C, 2014, PLOS ONE, V9, P0, DOI 10.1371/journal.pone.0102107, DOI	2014	5.38	**2016**	2018	
Ellingson BM, 2015, CURR NEUROL NEUROSCI, V15, P0, DOI 10.1007/s11910-014-0506-0, DOI	2015	4.89	**2016**	2020	
Patel AP, 2014, SCIENCE, V344, P1396, DOI 10.1126/science.1254257, DOI	2014	4.89	**2016**	2018	
Kickingereder P, 2016, CLIN CANCER RES, V22, P5765, DOI 10.1158/1078-0432.CCR-16-0702, DOI	2016	5.3	**2017**	2018	
Cui Y, 2017, EUR RADIOL, V27, P3583, DOI 10.1007/s00330-017-4751-x, DOI	2017	5.49	**2018**	2019	
Kickingereder P, 2016, RADIOLOGY, V280, P880, DOI 10.1148/radiol.2016160845, DOI	2016	4.81	**2018**	2020	
Shinohara RT, 2014, NEUROIMAGE-CLIN, V6, P9, DOI 10.1016/j.nicl.2014.08.008, DOI	2014	4.64	**2018**	2019	

Figure S2. Top 25 references with the strongest citation bursts in related publications.
