# Supplementary material for: Research on application of radiomics in glioma: a bibliometric and visual analysis
Source: Front Oncol. 2023 Sep 12;13:1083080. doi: 10.3389/fonc.2023.1083080 (PMC10523166; doi:10.3389/fonc.2023.1083080)
Supplement: Supplementary file 2 [file Table_1.pdf]

Table S1. Top 10 source journals for related publications

Rank	Count	Journals	IF 2022	Quartile in category
1	42	FRONTIERS IN ONCOLOGY	5.783	Q2
2	32	EUROPEAN RADIOLOGY	7.034	Q1
3	31	CANCERS	6.575	Q1
4	29	SCIENTIFIC REPORTS	4,996	Q2
5	13	JOURNAL OF NEURO-ONCOLOGY	4.506	Q2
6	13	NEURORADIOLOGY	2.995	Q3
7	11	JOURNAL OF MAGNETIC RESONANCE IMAGING	5.119	Q1
8	11	NEURO-ONCOLOGY	13.029	Q1
9	10	AMERICAN JOURNAL OF NEURORADIOLOGY	4.966	Q1
10	9	RADIOLOGY	29.146	Q1

Table S2. Top 10 keywords in the related publications

Rank	Count	Centrality	Year	Keywords
1	187	0.05	2014	glioblastoma
2	136	0.12	2012	survival
3	131	0.03	2015	classification
4	113	0.08	2014	magnetic resonance imaging
5	100	0	2018	machine learning
6	82	0	2017	tumor
7	79	0.02	2015	feature
8	66	0	2016	central nervous system
9	57	0.01	2013	idh
10	55	0.01	2017	radiomics
